# Discovering vesicle traffic network constraints by model checking

**DOI:** 10.1371/journal.pone.0180692

**Published:** 2017-07-06

**Authors:** Ankit Shukla, Arnab Bhattacharyya, Lakshmanan Kuppusamy, Mandayam Srivas, Mukund Thattai

**Affiliations:** 1 School of Computer Science and Engineering, Vellore Institute of Technology, Vellore, India; 2 Department of Computer Science and Automation, Indian Institute of Science, Bengaluru, India; 3 Chennai Mathematical Institute, Chennai, India; 4 Simons Centre for the Study of Living Machines, National Centre for Biological Sciences, Tata Institute of Fundamental Research, Bengaluru, India; Nankai University, CHINA

## Abstract

A eukaryotic cell contains multiple membrane-bound compartments. Transport vesicles move cargo between these compartments, just as trucks move cargo between warehouses. These processes are regulated by specific molecular interactions, as summarized in the Rothman-Schekman-Sudhof model of vesicle traffic. The whole structure can be represented as a transport graph: each organelle is a node, and each vesicle route is a directed edge. What constraints must such a graph satisfy, if it is to represent a biologically realizable vesicle traffic network? Graph connectedness is an informative feature: 2-connectedness is necessary and sufficient for mass balance, but stronger conditions are required to ensure correct molecular specificity. Here we use Boolean satisfiability (SAT) and model checking as a framework to discover and verify graph constraints. The poor scalability of SAT model checkers often prevents their broad application. By exploiting the special structure of the problem, we scale our model checker to vesicle traffic systems with reasonably large numbers of molecules and compartments. This allows us to test a range of hypotheses about graph connectivity, which can later be proved in full generality by other methods.

## 1 Introduction

Molecular interactions regulate the movement of cargo between different locations of eukaryotic cells. The hubs of this traffic network are large membrane-bound compartments known as organelles, between which cargo are transported within small vesicles [1–3]. The properties of compartments and vesicles are defined by the molecules they contain. Rothman, Schekman, and Sudhof were awarded the Nobel Prize for identifying key molecules involved in this process. These include Arf and Rab GTPases whose presence or absence encodes compartment identity [4, 5]; coat proteins that regulate cargo loading from source compartments onto vesicles [6, 7]; and SNARE proteins that regulate fusion of vesicles into target compartments [8, 9]. These molecules form the basis of an abstract representation of vesicle traffic [10, 11]: compartments are the nodes, and vesicles are the directed edges, of a transport graph; different molecules move in cyclical fluxes along this graph.

A detailed knowledge of how these molecules function is essential if we are to understand what kinds of traffic network topologies are physically possible. In particular, we focus on the transmembrane SNARE proteins, which form an essential part of the cellular addressing system. The fusion of a vesicle to a target compartment is driven by the zipping together of vesicle and compartment SNAREs [8, 9]. Typically a Q-SNARE triple-helix is confined to one membrane surface (e.g the compartment), while an R-SNARE single helix is confined to its fusion partner (e.g. the vesicle). When these two types of SNAREs are in close proximity they zip together into a four-helix bundle, forcing the vesicle to fuse with the compartment. There are many types of Q-SNAREs and R-SNAREs spread between different vesicles and compartments. Across eukaryotes, there are 20 broad SNARE varieties, though individual species can contain higher numbers (humans have 41) [12]. Only certain Q and R-SNARE combinations can zip into complexes, regulating precisely which vesicles fuse with which targets. For our analysis, we consider a Q-SNARE triple helix as a single complex and the R-SNARE as its cognate partner. We refer to zipping as SNARE pairing. Once a SNARE pairing drives a fusion event, the NSF protein uses the energy of ATP hydrolysis to split apart the four-helix bundle into its constituent Q and R components. Crucially, the zipping of a single active SNARE complex on a vesicle with a single active SNARE complex on a compartment is necessary and sufficient for fusion [13]. This fact alone places stringent constraints on how SNAREs can move through the traffic system. It must be the case that SNARE zipping can be inactivated by regulatory molecules: without this, a vesicle SNARE that just participated in an incoming fusion event would also cause all outgoing vesicles carrying the SNARE to fuse back. Molecules such as Rabs, Arf GTPases, and Sec/Munc proteins on vesicles and compartments can activate or inhibit SNAREs; SNARE longin domains are sites of known regulatory activity [4, 14]. However, the intricacies of SNARE regulation are largely unknown. In the absence of such information, how can we work toward a predictive understanding of vesicle traffic?

Here we take an abstract computational approach toward this goal, informed by biological detail. We do highly efficient exhaustive searches over the space of allowed vesicle traffic network topologies, using computational tools called model checkers. These tools are central to formal verification methods in computer science, and are increasingly being applied to understand biological systems, including gene regulatory networks [15–17]; ours is the first application of model checking to vesicle traffic networks. We determine all possible topologies of steady-state vesicle traffic networks that are consistent with models of SNARE regulation. We study a hierarchy of regulation models of increasing complexity, and show that not all directed graphs can represent the transport graph of a physically realizable vesicle traffic network. This analysis makes several explicit predictions about how SNAREs and their regulators are transported, used, recycled and re-used across a cell.

## 2 Results

### 2.1 The model

On timescales of minutes, the following assumptions reasonably capture the important aspects of the Rothman-Schekman-Sudhof (RSS) model [2] of vesicle traffic.

1. A cell is a set of compartments exchanging vesicles.
2. Compartments are neither created nor destroyed.
3. Each compartment is in steady state, gain and loss balance.
4. Molecules are neither created nor destroyed.
5. Molecules move via vesicles of uniform size.
6. Identical vesicles have identical target compartments.
7. Fusion of vesicles to compartments is driven by specific SNARE pairing.
8. The activity of a SNARE can be regulated by other molecules present on the same compartment or vesicle.
9. An active SNARE pair is necessary and sufficient for fusion.

**Fig 1:**
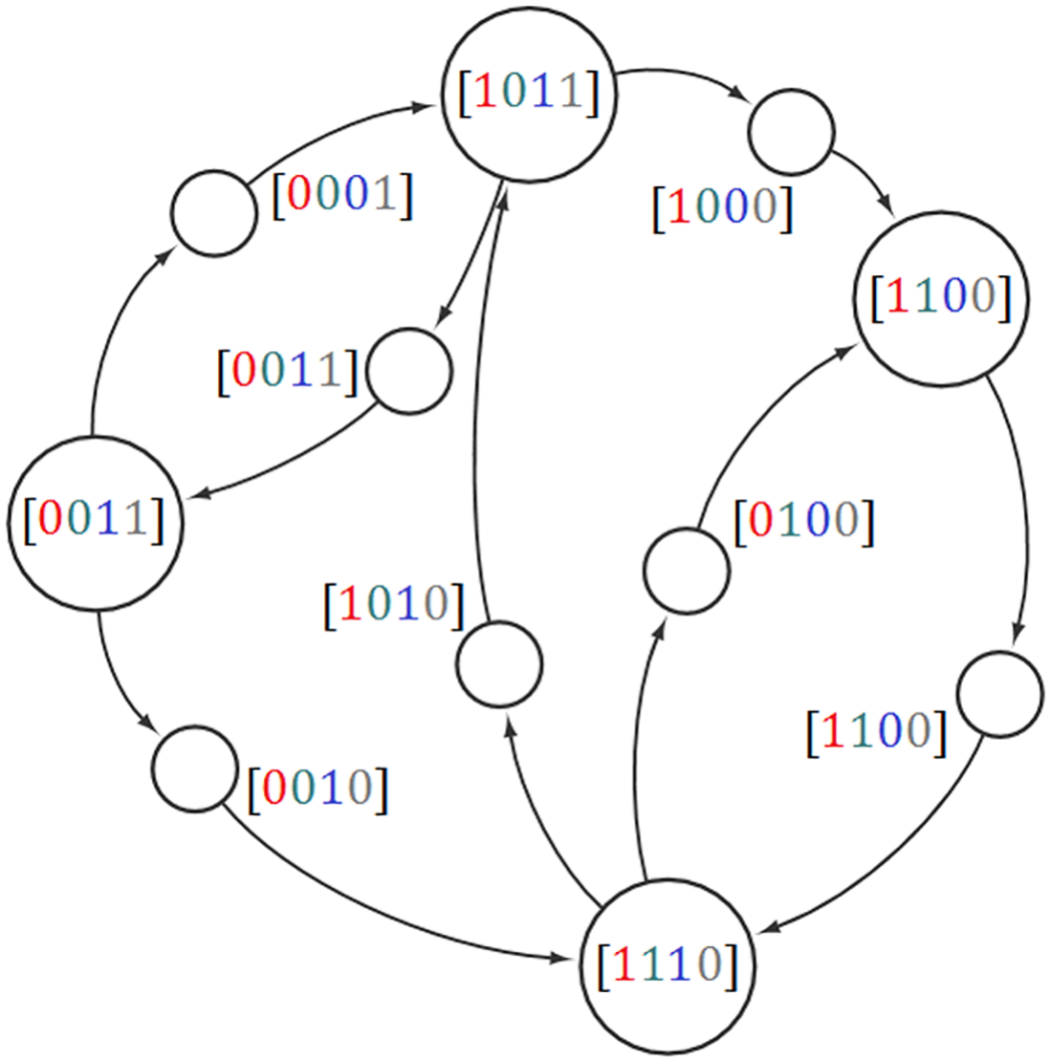
The vesicle traffic graph in steady state. Nodes (large circles) are compartments; vesicles (small circles) are associated with directed edges. The binary labels represent the presence/absence of four molecular types in this example. The actual amounts of these molecules on each compartment, and actual fluxes along each vesicle edge, can be positive real numbers. By construction, the total incoming flux can be made to balance the total outgoing flux of each molecular type at every node. Note that no pair of vesicles has identical compositions, yet all molecular components move in closed cycles. This is related to the fact that this graph is 3-connected (see section 2.2).

Let us assume there are M molecular types in the system. These include SNARE proteins, but also include their regulators, and structural molecules such as lipids. Each compartment is a node, specified by an M-length vector giving the amount of various molecular types it contains. Each vesicle is an edge connecting one compartment to another, specified by an M-length vector giving the flux of each molecular type. For this graph to be in steady state, the total incoming and outgoing flux of each molecular type must balance at every node (Fig 1). We assume throughout that self-edges are not permitted (biologically, this could be justified by efficiency considerations).

**Fig 2:**
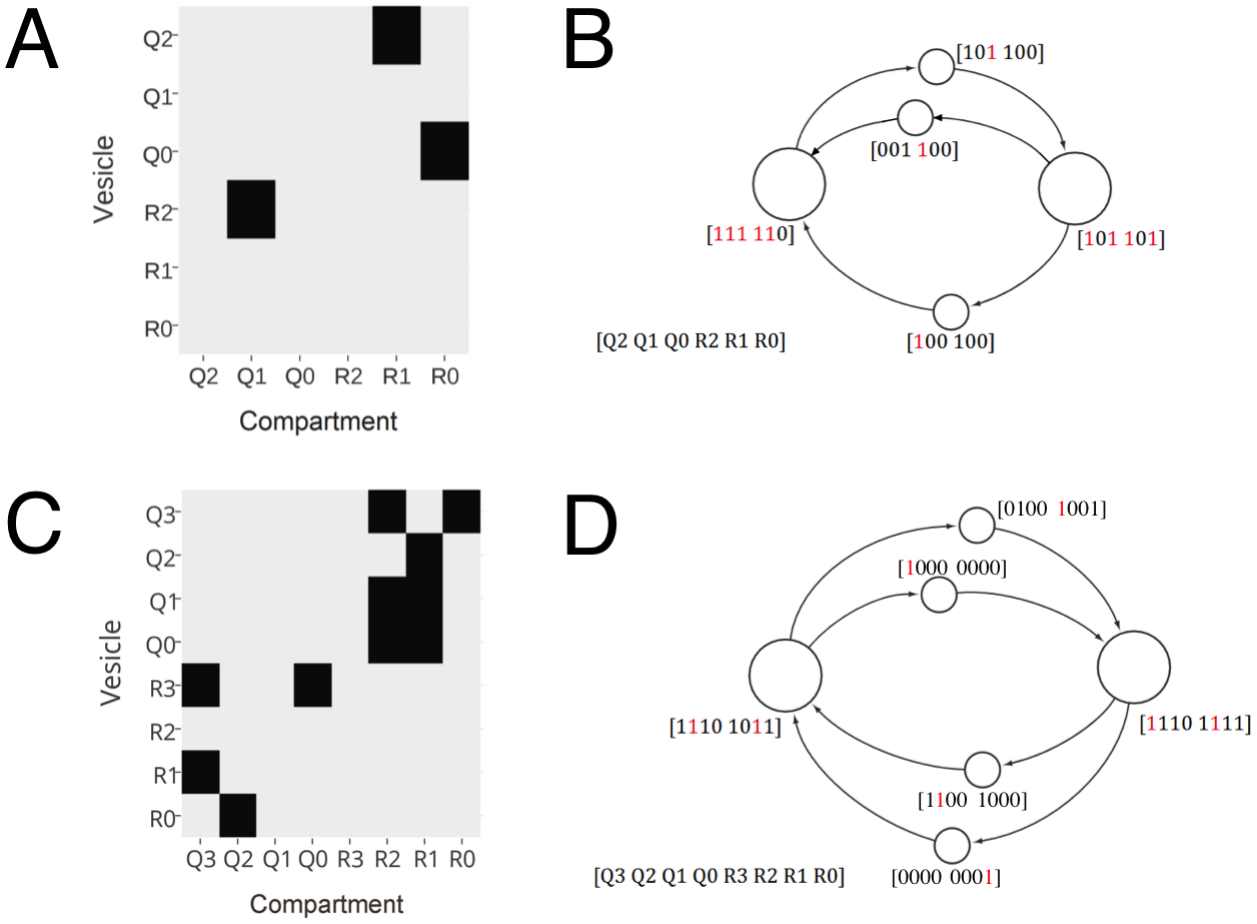
SNARE pairing matrices and the resulting steady state vesicle traffic networks. SNARE interactions generate the vesicle traffic network. (A,C) Examples of SNARE pairing matrices. Q0, Q1, etc. are Q-SNAREs, R0, R1, etc. are R-SNARES. Dark squares represent SNARE pairings that result in fusion. Each column represents a SNARE type on compartments (nodes), and each row represent a SNARE type on vesicles (edges). SNAREs can be active or inactive depending on the other molecules present on the same vesicle or compartment. If at least one active Q-SNARE type on one membrane interacts with at least one active R-SNARE type on the opposite membrane, compartment-vesicle fusions will occur. (B,D) Steady state vesicle traffic networks governed by SNARE pairings. Nodes (large circles) are compartments; vesicles (small circles) are associated with directed edges. As in Fig 1, we only show the presence or absence of each molecular type on each compartment or vesicle: the first half of the binary vector represents Q-SNAREs, and the second half represents R-SNAREs. The actual fluxes can be positive real numbers, values of which can always be found in order to keep the system in steady state. Active SNAREs are shown in red, inactive SNAREs in black. (A,B) The case of Table 1 row 2, where there is no regulation on compartments (all SNAREs are active) but there is Boolean regulation on vesicles (so only those SNAREs shown in red are active). The necessary and sufficient condition for this case is a 3-connected graph (see section 2.4). (C,D) The case of Table 1 row 3, where SNAREs on compartments have Boolean regulation, and SNAREs on vesicles are regulated by SNARE-SNARE inhibition (i.e. if pairing-compatible SNAREs exist on the vesicle, they neutralize one another). The necessary and sufficient condition for this case is a 4-connected graph (see section 2.4). We have only shown very simple two-compartment examples here, but our results on graph connectedness are exhaustive, and carry over to much more complex cases.

We next have to specify precisely how SNAREs prefer to pair with one another, and how the activity of these molecules might be regulated by other molecules in the system. We assume M/2 Q-SNARE types and M/2 R-SNARE types. Fusion is defined by an *M* × *M* binary SNARE pairing matrix, where each non-zero entry states that the corresponding Q-SNARE and R-SNARE pair is fusion competent (Fig 2A and 2C). The rows/columns represent SNAREs on vesicles/compartments respectively. There are only non-zero entries for Q-R or R-Q pairs. We assume that, due to size and curvature differences, the behavior of molecules on vesicles can be distinct from their behavior on compartments; the matrix is therefore not necessarily symmetric. Rather than invoking further molecular types, we assume the most general situation in which each molecule can take on SNARE-based fusion roles as specified above, or additional regulatory roles. Non-SNARE molecules in this format are those that do not participate in fusion (have no corresponding non-zero entries in the SNARE pairing matrix) but do influence the activity of other SNAREs as described below. Molecules that are involved in both fusion and regulation could represent two real molecular types that are always co-localized. This representation is therefore without loss of generality.

We study a hierarchy of models for varying degrees of regulation of SNARE activity (Table 1). In the simplest case, each SNARE is always considered to be constitutively active (no-regulation model). In the most complex case, the activity state of each SNARE could depend on the presence or absence (defined by a logical Boolean function) of all the other molecules on the corresponding vesicle or compartment (Boolean regulation model). Intermediate between these cases are those in which activity is regulated only on the compartment, or only on the vesicle. We also consider a special case, in which regulation on vesicles has a very restricted form. In this case, we assume that if a fusion-competent Q-R SNARE pair is present on a vesicle (i.e. a pair with a non-zero entry in any part of the SNARE pairing matrix) then both molecules are rendered inactive. This is motivated by the fact that these two molecules are likely to zip together, and that NSF is not present on vesicles to unzip them. We refer to this version of regulation as SNARE-SNARE inhibition.

**Table 1:** SNARE regulation and graph connectedness.

Sr.No	Regulation on compartment	Regulation on vesicle	Required graph connectivity	
			Necessary	Sufficient
1.	Boolean function	Boolean function	2-connected	3-connected
2.	None	Boolean function	3-connected	3-connected
3.	Boolean function	SNARE-SNARE inhibition	4-connected	4-connected
4.	None	SNARE-SNARE inhibition	No graph	No graph
5.	Boolean function	None	No graph	No graph
6.	None	None	No graph	No graph

### 2.2 Graph connectedness as an informative feature

For molecules moving along a directed graph, each type of molecule must move in a cycle for each node to be in steady state. This requirement is equivalent to the transport graph being strongly connected [18]: there is a directed path from every node to every other node in the graph (e.g. Fig 1). We restrict our discussion to transport graphs with a single strongly connected component; equivalently, our statements separately apply to each connected component of a graph with multiple components. Given a directed graph, the underlying undirected graph can satisfy various degrees of connectedness. An undirected graph is said to be k-edge-connected (here referred to simply as k-connected) if a minimum of k edges must be removed to disconnect the graph. A strongly connected graph must be at least 2-connected since each set of nodes that has an incoming vesicle must also have an outgoing vesicle. But this level of connectedness may not be sufficient to satisfy molecular constraints. For example, if SNAREs are completely unregulated and thus always active, they cannot be used to generate a traffic network (Table 1, bottom row). If these SNAREs are regulated by relatively simple rules (Table 1, SNARE-SNARE inhibition) then only highly connected traffic networks can be generated using them. Finally, if SNAREs can be regulated extremely flexibly so each SNARE’s activity is a Boolean function of compartment or vesicle composition, then many more networks, each of much lower minimal connectivity, can be generated. In short: by allowing SNARE regulation to be more flexible, we can enable more types of graph topologies to be realized.

### 2.3 The model checking approach

#### 2.3.1 Combinatorial explosion

The analysis of vesicle traffic systems is a difficult problem because of the combinatorial scaling of possible traffic topologies and regulatory rules. For example, we might want to check some conjecture of interest for all 3-connected graphs and all possible variations of SNARE regulation rules. The number of graphs of specified connectivity grows exponentially with the number of nodes: Table 2 shows how many 3-edge-connected graphs [19] exist (without parallel or self edges) as node number N increases. The complexity scales even faster with molecule number [10]. Suppose there are M types of molecules in the system, and therefore 2^*M*^ possible compositions of compartments or vesicles. There are then 2^2^*M*^^ Boolean function over this space, each of which might be applied to regulate each of the M SNAREs.

**Table 2:** Number of simple 3-edge-connected unlabeled N-node graphs.

N	Total Number of graphs
1.	0
2.	0
3.	0
4.	1
5.	2
6.	15
7.	121
8.	2159
9.	68715
10.	3952378

#### 2.3.2 Model checkers

**Fig 3:**
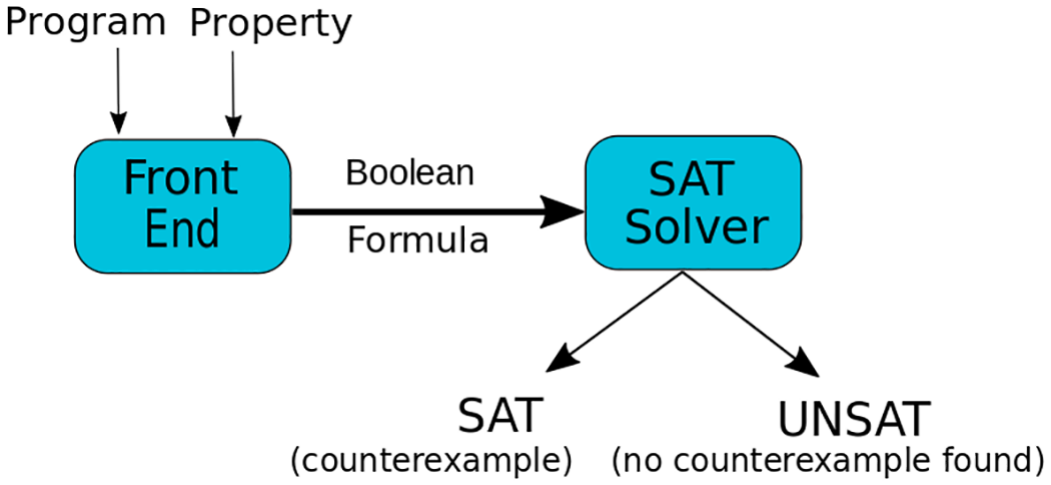
An overview of model checkers and CBMC as a tool. CBMC’s front end (CFE) converts a program and a property into a Boolean formula which is then verified using a SAT solver. CBMC will produce a counterexample in the case of violation of the property.

Previous analyses of vesicle traffic networks dealt with this combinatorial explosion by using a sampling approach [10, 11]. In these analyses, vesicle traffic is modeled as a dynamical system. The traffic rule specifies how the system transitions from one time point to the next. Given a traffic rule and an initial condition, the system is evolved over time until a steady state is reached. By studying a large sample of randomly generated traffic rules, such analyses can make statistical claims about vesicle traffic. In contrast, here we seek understand properties of vesicle traffic networks over all possible traffic rules, not just for a sampled subset. We would also like to directly make statements about steady state properties, rather than using transition dynamics to first search for steady states. Formal verification tools such as model checkers serve precisely this purpose. The first step of formal verification is to encode the vesicle traffic problem symbolically. Once this is done, various steady-state properties can be checked directly, without explicit enumeration of traffic rules or graph structures (Fig 3).

Here we use model checking to explore how assumptions of the RSS model constrain the structure of the vesicle transport graph. More precisely: we check a specified logical property on every possible state of a model constructed using variables ranging over Boolean or any finite discrete type. We encode our question as a Boolean satisfiability (SAT) problem whose variables represent: the structure of the transport graph, the presence or absence of various molecules on each compartment and vesicle in the graph, and the rules of SNARE pairing and SNARE regulation. The logical property checked asserts whether the rules of the RSS model hold, assuming a certain degree of graph connectedness also holds. Modeling the problem as a SAT instance has the advantage of automatically checking some constraint of interest for all possible graphs of up to a certain size, for all possible variations of molecular interactions, thus boosting our confidence in the property verified. We find that the model checker approach, with a few additional workarounds, scales reasonably well for studying the vesicle traffic network. More details on our implementation are provided in Methods. Interestingly, in the case some property of interest is violated, model checkers provide a counterexample, which is often useful in further developing biological hypotheses.

### 2.4 Graph connectedness under different models of SNARE regulation

There is a non-trivial coupling between the steady state condition and the SNARE pairing and regulation rules. If we are given these rules ahead of time, it is highly unlikely that a given graph will be a possible steady state of a vesicle traffic system. To formalize this we introduce the notion of *necessary* and *sufficient* conditions regarding k-connectedness of the underlying graph for a transport network to meet steady state requirements:

*Necessary Condition*: k-connectedness is said to be a necessary condition for a vesicle transport network model if k-connectedness is the least possible graph structure for a steady state network, over all possible SNARE pairing and regulation rules of the appropriate variety.

*Sufficient Condition*: k-connectedness is said to be a sufficient condition for a vesicle transport network model if every k-connected graph is guaranteed to attain a steady state, for at least one SNARE pairing and regulation rule of the appropriate variety.

A summary of our results, based on running the model checker for graphs with up to N = 8 nodes and M = 32 molecules, is presented in Table 1. No matter what the rules of molecular interactions and regulation, steady state requires the graph structure to be at least strongly connected, and therefore 2-connected. Beyond this, we find that 3-connectedness is necessary and sufficient for the Boolean regulation model on vesicles (Fig 2A and 2B), while 4-connectedness is necessary and sufficient for the more constrained SNARE-SNARE inhibition on vesicles and Boolean regulation on compartments (Fig 2C and 2D). For more constrained regulation models (including the case where SNAREs are always active) we did not find any possible graphs representing steady state vesicle transport networks. This strongly suggests (but does not prove, since we only checked graphs of finite size) that no such graphs exist. Once we have confidence in these patterns, these results may be rigorously proved by more general mathematical arguments.

## 3 Conclusion

We have established a connection between vesicle transport graph connectedness and underlying rules of SNARE pairing and regulation. Our results contain many predictions which can in principle be tested through cell-biological experiments. However, our current knowledge of the actual vesicle transport graph of a cell is very poor: it is likely that there are many vesicle transport routes that are still uncharacterized. Even in those instances where vesicles are known to exist, the precise molecular drivers of their creation and fusion are often unknown. Therefore, a direct verification of our predictions based on graph connectedness alone is not likely. However, more indirect predictions based on how SNAREs are regulated can be explored. In this context, the Sec/MUNC proteins or SNARE longin domains are interesting targets of study [13, 14].

We have also learned important lessons about the applicability of the model checking approach for biological analysis. While the use of CBMC and SAT solvers for analyzing vesicular transport networks proved to be quite effective, these tools are limited in the size of systems they can automatically handle. For example, we were able to analyze systems with up to 8 compartments and 32 molecules. This is comparable to the SNARE homolog number and compartment number of a typical eukaryotic cell [12]. Scaling beyond this size would be a challenge for current state-of-art solvers. It would be interesting to explore the use of more advanced methods, such as word-level SMT solvers [20] and semi-automatic inductive techniques for analyzing systems of arbitrary sizes. More broadly, we see our approach as a way of generating strong and testable hypotheses over a useful range of system sizes, which can later be tackled using more general methods.

Though we have not discussed functional aspects here, it is important to remember that the vesicle traffic system exists to move molecules from sites of production to sites of use, to modify these molecules as they move, and to exchange material with the extracellular environment. If the molecular constraints were so limiting that drastic changes to traffic systems could never occur, cells would not be able to adapt to new functional requirements. However, our results suggest the opposite: that once the basic molecular mechanism of vesicle traffic is established, cells are easily able to explore a variety of graph topologies over evolutionary timescales. The space of 3-connected or 4-connected graphs is vast, allowing a broad diversity of vesicle traffic in eukaryotic cells, reflecting the distinct environmental niches within which they operate.

## 4 Methods

### 4.1 CBMC

The model checker tool that we have used is CBMC [21, 22], a “bounded model checker (BMC)” for C-programs. Unlike a general model checker [23], BMC checks the verified property for all the possible states, i.e., assignments of variables in the model, up to a certain “depth,” a parametric limit on the size of the model. CBMC allows the depth of model to be incrementally increased to any desired value.

CBMC (Fig 3) consists of a C-language front-end (CFE) and SAT Solver back-end (SAT). CBMC accepts ANSI-C programs interspersed with special annotations (called *assumes* and *assertions*) to express constraints on the model and properties to be checked. In our exercise, the vesicle transport network was modeled as a non-deterministic C-program manipulating a graph with labeled edges with the fusion rules and steady state properties expressed as constraints using *assumes*. The correspondence relationship between connectedness conditions and guarantees for steady state were expressed as *assertions* to be checked. Given an exploration depth provided by the user, which in our case corresponds to the size of the graph, CBMC verifies the properties by executing the following steps:

1. Convert (using CFE) the model and the properties into a Boolean formula (verification condition) such that the property is true of all behaviors of the model up to the specified depth iff the Boolean formula is valid.
2. Check (using a SAT solver) validity of the verification condition. CBMC reports successful verification if the formula is found to be valid. If not, then CBMC produces a counterexample, an assignment to the variables of the model, that is a witness to the violation of the property.

The way CBMC works is as follows: for a given property, CBMC checks its validity by trying to find a satisfiable assignment to the negation of the property. To assert that property P is valid, CBMC will check for a satisfiable assignment of the formula ¬*P*. CBMC has a built-in SAT solver called MiniSat [24], but it is also possible to use various other SAT solver black-boxes for property verification. Besides using MiniSat as a default SAT solver for our model, we have used different SAT solvers for the verification of the property, particularly CryptoMiniSat [25] which was the winner of SAT 2015 Competition [26]. MiniSat performed satisfactorily in comparison to other SAT solvers.

### 4.2 Encoding the Rothman-Schekman-Sudhof model in CBMC

#### 4.2.1 Expressing the conjecture as assertions

The conjecture relates graph-connectedness to a particular variation of rules of SNARE pairing and regulation of the vesicle traffic system. We have to find what is the least structural connectedness of the transport graph required for the system driven by a particular set of rules (necessary condition) and the minimum structural condition that ensures every transport graph with that fundamental structure satisfies the stated set of rules (sufficient condition). We express both these conditions as assertions in our program.

#### 4.2.2 Checking necessary conditions

In our program, we represent the steady state condition, SNARE pairing and regulation rules, and the connectedness property that the system needs to follow, as constrained Boolean functions on graphs (G) and on labeled edges and nodes (a Boolean function f). Each of these constrained functions is defined to be TRUE for a G and f: if and only if the corresponding condition holds for the given G and f. We use these functions to define the assertion that characterizes the necessary condition to be checked. We will show in later sections how we encode these Boolean functions.

To ensure that k-connectedness is a necessary condition, we have to find a k-connected graph that satisfies all the rules (and constraints) of the system and no graph with less connectivity that does that. This is expressed in two parts—the first past (Listing 1) that shows existence (SAT check) of a k-connected graph and second part (Listing 2) that shows non-existence (UNSAT check) of any k-1 connected graph that satisfy the required conditions. Let’s fix our conjecture: “4-connectedness is a necessary condition for the system regulated by a Boolean function on the node and SNARE-SNARE inhibition on the edge”. To find a 4-connected graph we specify our property as a query for the existence of a Boolean function f such that there is a satisfiable assignment to the conjunction of our Boolean variables: C1, C2, C3, C4 (refer Listing 1). Symbol # is used to show comments in the listings.

**Listing 1. Necessary condition property**

# C1: Steady state condition.

# C2, C3: Fusion rules.

# C4: Graph is 4–connected.

# G: Graph, f: Constrained function.

# There exists a 4 connected graph for the given model.

SAT (C1(G) ∧ C2(G, f) ∧ C3(G, f) ∧ C4(G))

Second, we have to check the possibility of any 3-connected or 2-connected graph to have a satisfiable assignment. For this part, we just change Boolean variable C4 to C34′ to represent graphs that are 3-connected but not 4-connected. If there exists a satisfiable assignment for any such graphs, the model checker will provide a counterexample and a witness to the violation of the conjecture.

**Listing 2. Necessary condition property**

# C1: Steady state condition.

# C2, C3: Fusion rules.

# C34′: Graph is 3–connected but not 4–connected.

# G: Graph, f: Constrained function.

# There exists no 3–connected graph for the given model.

UNSAT (C1(G) ∧ C2(G, f) ∧ C3(G, f) ∧ C34′(G))

#### 4.2.3 Checking sufficient conditions

Similarly, for the conjecture that 4-connectedness is a sufficient condition for the system regulated by Boolean regulation on the node and SNARE-SNARE inhibition on the edge, we specify the property as: for every 4-connected graph, there exists a satisfiable assignment following the rules of the system as shown in Listing 3. Note that the specification of this requires quantifier alternation. Most SAT solvers can check (at least efficiently) only quantifier-free formulas. To handle this challenge in CBMC, we used a combination of nondeterminism and Boolean enumeration at the C-source level to eliminate quantifiers, as explained further.

**Listing 3. Sufficient condition property**

# C1: Steady state condition.

# C2, C3: Fusion rules.

# C4: Graph is 4–connected.

# G: Graph, f: Constrained function.

# ⊃: Material conditional (if .. then), ∧: Logical and.

# ∀: Universal quantifier, ∃: Existential quantifier.

# For every 4–connected graph following conditions must be valid.

VALID (∀ G: C4(G) ⊃ (∃ f: C1(G) ∧ C2(G, f) ∧ C3(G, f)))

#### 4.2.4 Encoded model

- We use a two-dimensional array to represent the transport graph, where *graph*[*i*][*j*] = *x* if there are exactly x edges between node i and j. We leave the graph completely arbitrary and constrain its structure to be k-connected. The Boolean variable C4 is used to build up this constraint. We use this variable as a part of the property to make sure that counterexample must be a k-connected graph and hence relate it with the biological rules and constraints.
  Upfront we fix the total number of distinct molecules (M) and tag each node with a bitvector of this length, where first half represents Q-SNAREs and rest are R-SNAREs (Fig 2). The bit vector provides information about the presence/absence of the ith molecule by checking whether the ith bit is on (1) or off (0). E.g. for a vesicle labeled [111 110], SNAREs Q2 Q1 Q0 (111) and R2 R1 are present, but SNARE R0 is absent (110).
- An edge represents a vesicle going from the source to fuse to the target compartment. We have to make sure that only a subset of molecules present on a source node are allowed to go out. E.g. the first edge of Fig 2B has label [101 100] representing SNAREs Q2, Q0 and R2 going out on the vesicle, which is the subset of its source compartment labeled [111 110]. SNARE pairing and regulation rules dictate the validity of the edges.
- In this abstract model, every molecule behaves either as a Q-SNARE or R-SNARE, and as a constraint we do not allow self-edges in the graph.

We allow multiple edges between two nodes but restrict this to be at most two to keep the search space of graphs to be explored within a reasonable limit for SAT solvers. In CBMC this is done by using __CPROVER_assume statement as shown below, which specifies nedges to be an arbitrary integer, i.e., nondetuint(), but constrained to be between 0 and 2.

**Listing 4. Multiple edges between two nodes**

unsigned int nedges = nondet_uint ()

__CPROVER_assume(nedges ≥ 0 ∧ nedges ≤ 2)

We already know that graphs with too few edges will not meet the criteria due to the steady state condition, and similarly, a graph with too many edges might trivially fulfill the criteria. Hence we use the model checker to check graphs starting with a small edge count (E) and incrementally increasing it after that.

### 4.3 Steady state condition

#### 4.3.1 Steady state specification

One significant restriction in our model is the steady state condition or homeostasis requirement for the whole system. It means that each molecule leaving the node on a vesicle should come back to its source node in a cycle, i.e., for every molecule leaving the node there exists a cycle with that molecule present on each of the edges and nodes of the path taken. When such a path exists, the flux along that cycle can be an arbitrary real number. The flux of any SNARE across any vesicle edge is given by the sum of the fluxes of each cycle that edge is a member of (Fig 1). Listing 5 shows a sketch of the steady state specification described above.

**Listing 5. Steady state specification**

# Every edge E is represented by a label; labelE (a bitvector).

# E(x, y): Edge between node x and y. E. source = x, E. target = y

# labelE (m): mth molecule is present on edge E.

# Edge (m) (x, y): Edge between node x and y with labelE (m).

# seq (a1 .. an): Each ak represents a node. len(seq) is |seq|.

# N: Total number of nodes, =: Mathematical equality.

∀ z ∈ {Nodes} ∀ E ∈ {Edges}: E. source = z ⊃

  ∀ m ∈ {Molecules}: labelE (m) ⊃

    (∃ a seq (a1 .. an): (2 ≤ |seq| ≤ N) ∧

      ((a1 = z) ∧ (a2 = E. target) ∧

        ((∀ k from 1 to |seq| − 1) ⊃ Edge (m) (ak, ak+1)) ∧

        (Edge (m) (an, a1)))

#### 4.3.2 Steady state encoding

It is evident from the specification (Listing 5) that to encode the steady state condition we require quantifier alternation (∀ .. ∃). But CBMC currently supports only quantifier-free specifications. To get the effect of quantifier alternation we use a subtle technique involving a mixture of enumeration and the power of nondeterminism. The intuition behind this is simple. We use an array variable to represent an arbitrary length cycle, where each element of this array is a node, forming a sequence of node hops taken by the molecule; this is a route the molecule could adopt to get back to its source node. During the actual check with SAT solver, the model checker will find this cycle.

We initialize Boolean variable C1 to TRUE and use this variable to make sure that further checks are compatible with the chosen cycle. We check that there is an edge between each of the selected cycle sequence, with that particular molecule present on each of the edges. C1 stays TRUE if the checks are valid and in the case of any compatibility violation it gets turned off. To make sure that this holds for every molecule going out on a vesicle, we use this variable as a part of our property.

### 4.4 Fusion rules

#### 4.4.1 Fusion rule specification

Our fusion rules consist of two different mechanisms. First: A SNARE pairing mechanism which determines compatible Q-R pairs on vesicles and compartments that can cause fusion. Second: regulatory mechanisms on edges and on nodes (possibly distinct) which regulate SNARE activity based on the presence/absence of other molecules on the corresponding node or edge. For an edge to be valid, at least one SNARE pair on the vesicle and target compartment must be active, and have a non-zero entry in the pairing matrix (Listing 6). We enforce that two edges of identical composition should go to the same target (Listing 7). We have to ensure that fusion respects the graph structure by the vesicle (edge) under consideration, fusing only with its target node and not with any other node (Listing 8).

**Listing 6. Edge respects fusion rules**

# Make sure that every present edge follow fusion rules.

# E(x, y): Edge between node x and y.

# Activity of molecules on edge and nodes is determined by a boolean function.

# ActiveE (i): ith molecule is active on edge E.

# ActiveY (j): jth molecule is active on node y.

# M: Total number of distinct molecules.

# pairingMatrix: SNARE pairing matrix.

∀ x, y ∈ {Nodes}: E(x, y) ⊃

(∃i, j ∈ [1 ..M]: ActiveE (i) ∧ ActiveY (j)

            ∧(pairingMatrix (i, j) = 1))

**Listing 7. Two edges of identical composition should go to the same target**

# labelE: Label of edge E.

# E(x, y): Edge between node x and y, where E. source = x, E. target = y.

(labelE = labelE′) ⊃ (E. target = E′. target)

**Listing 8. Graph topology is respected**

# E(x, y): Edge between node x and y.

# Activity of molecules on edge and nodes is determined by a boolean function.

# ActiveE (i): ith molecule is active on edge E.

# ActiveZ (j): jth molecule is active on node z.

∀ x, y, z ∈ {Nodes}: E(x, y) ⊃

(∀i, j ∈ [1 ..M] (ActiveE (i) ∧ ActiveZ (j) ∧

       pairingMatrix (i, j) = 1) ⊃ (z = y))

#### 4.4.2 Encoding fusion rules

To encode fusion we use a SNARE pairing matrix (an *M* × *M* matrix, Fig 2A and 2C). To encode regulatory mechanisms we use an arbitrary Boolean function for each SNARE (active SNAREs represented in red in Fig 2B and 2D). We use different functions for two different regulatory mechanisms; one on the edges and another on the nodes. In one special variation of regulation (SNARE-SNARE inhibition) in place of using Boolean regulation functions we use the SNARE pairing matrix itself to determine inhibition of SNAREs on the edges.

Here we show the whole mechanism by use of an example. Let us fix a variation of the model (Table 1, row 2): vesicle traffic system with Boolean regulation only on edges; i.e. every molecule present on a node is active (Fig 2A and 2B). We specify an *M* × *M* matrix SNARE pairing matrix (Fig 2A). Consider the first vesicle leaving the node on the left, with the label [101 100] (i.e. the vesicle carries SNARES Q2, Q0, and R2). Due to the Boolean regulatory function, SNAREs Q2 and R2 are inactive (the Boolean functions themselves are not shown). We will leave the choice of the Boolean function for each molecule on the edge to be arbitrary, and the model checker will fill this gap while building a counterexample in case of a violation of the logical property. Only the Q0 SNARE is active (red), and according to the SNARE pairing matrix, it has to find its corresponding SNARE R0 on the target node in the active state. This is precisely the case, causing fusion. We use Boolean variable C2 to make sure that every edge follows the fusion rules and use C3 to ensure that a vesicle fuses only with its target compartment and not with any other compartment.

### 4.5 Encoding the sufficient condition property

Specifying the sufficient property again requires quantifier alternation (Listing 3) but unlike the encoding specification case, using nondeterminism and enumeration directly is not possible for defining properties in CBMC. So we have few prospects:

1. Generate all possible graphs of a particular size by cleverly using constrained nondeterminism and array unwinding features of CBMC.
2. Convert the model and specification to Quantified Boolean Formulas (QBF) and use QBF SAT solvers [27].
3. Use a combination of 1 and 2.

In this work, we used the first option by using the CBMC C front-end and SAT solver to generate all possible graphs of a certain size, say 5, and increasing this incrementally as much as possible. We believe this method is still better than checking by simulation as it does not enumerate every graph individually at the source level but uses the search capability of SAT solvers for performing part of the enumeration.

Alternating quantifiers are required for modeling and specifying properties of complex biological systems. SAT solvers work most efficiently only for quantifier-free formulas. Since we were working with bounded size graphs, we eliminated quantifiers by a combination of enumeration and use of nondeterminism, which is cumbersome and not optimal. It would be interesting to investigate the impact of using advanced techniques for quantifier elimination [28] in SAT solvers and model-checkers for applications such as ours. Recently CBMC has been extended to support limited forms of quantifiers in its specification language and translation to SAT for arrays implemented in an experimental version of the tool. This uses combinations of model enumeration and QBF techniques, we should explore how well these features work for our application.
